# Brief memory reactivations enable generalization of offline visual perceptual learning mechanisms

**DOI:** 10.1038/s41598-025-06564-y

**Published:** 2025-07-01

**Authors:** Taly Kondat, Yuka Sasaki, Takeo Watanabe, Nitzan Censor

**Affiliations:** 1 Sagol School of Neuroscience, Tel Aviv University, 69978 Tel Aviv, Israel; 2 School of Psychological Sciences, Tel Aviv University, 69978 Tel Aviv, Israel; 3 Department of Cognitive and Psychological Sciences, Brown University, Providence, USA

**Keywords:** Neuroscience, Cognitive neuroscience, Perception

## Abstract

Perceptual learning can significantly improve visual sensitivity even in fully matured adults. However, the ability to generalize learning to untrained conditions is often limited. While traditionally, perceptual learning is attributed to practice-dependent plasticity mechanisms, recent studies suggest that brief memory reactivations can efficiently improve visual perception, recruiting higher-level brain regions. Here we provide evidence that similar memory reactivation mechanisms promote generalization of offline learning mechanisms. Human participants encoded a visual discrimination task with the target stimulus at retinotopic location A. Then, brief memory reactivations of only five trials each were performed on separate days at location A. Generalization was tested at retinotopic location B. Results indicate remarkable enhancement of location B performance following memory reactivations, pointing to efficient offline generalization mechanisms. A control experiment with no reactivations showed minimal generalization. These findings suggest that reactivation-induced learning further enhances learning efficiency by promoting offline generalization mechanisms to untrained conditions, and can be further tested in additional learning domains, with potential future clinical implications.

**Supplementary Information:**

The online version contains supplementary material available at 10.1038/s41598-025-06564-y.

## Introduction

Visual perception is critical for daily function and can be remarkably enhanced even in fully developed adult humans through the process of perceptual learning, resulting in an improved ability to detect, discriminate, and recognize visual information^1–9^. While such perceptual learning changes are long-lasting^10,11^, they are often limited in their ability to be generalized to untrained stimuli in similar settings and tend to be highly specific to the trained stimulus features (e.g. orientation, and retinotopic location^7,12–16^).

Typically, perceptual learning requires repetitive exposure to visual stimuli^1,17–21^. Consistently, it is attributed to practice-dependent neural plasticity mechanisms^3,22^, which have been predominantly documented in early visual brain regions^11,12,23,24^. These practice-dependent mechanisms have been associated with the distinctive specificity previously documented in perceptual learning, as neural coding in early visual brain areas is commonly specific to low-level visual features^25^. Over the last decade, additional studies have pointed to potential mechanisms that may enable generalization of perceptual learning^26–33^. Thus, accumulating evidence indicates that perceptual learning can engage higher-level cortical regions^12,34–42^.

A plethora of studies originating from fear-conditioning in rodents and extending to additional forms of learning and memory including in humans, have provided evidence suggesting that reactivation of a previously consolidated memory opens a time window for its modulation^43–54^. Such reactivation mechanisms may enable degradation, strengthening, or updating of existing memories. Recent studies have demonstrated that brief exposures to a previously encoded perceptual task enable perceptual learning by potentially triggering offline memory reactivation-reconsolidation cycles^47,49,55^. This reactivation-based learning was shown to recruit greater engagement of higher-level brain regions such as the intraparietal sulcus and the precuneus^55^. Unlike early visual areas, these higher-level brain regions are not specific to basic stimulus features, raising the hypothesis that, in parallel to other mechanisms, brief memory reactivations may also facilitate offline generalization mechanisms of perceptual learning.

To address this question, we leveraged the reactivation-induced learning paradigm^49^, replicated in Kondat et al.^55^. In this paradigm, human participants encode a visual texture discrimination task, then the memory is reactivated on separate days, and performance is tested on the final day of the study. Similar to standard learning paradigms in the texture discrimination task^10–12,37,56–58^, offline learning is evaluated as the between-session improvement in discrimination thresholds between the encoding and the test sessions^49,55^. Therefore in the current study, to evaluate generalization of these learning mechanisms, the final test was performed at a novel, untrained retinotopic location, and offline improvement between the encoded and the novel location was measured. Accordingly, participants initially encoded a visual discrimination task with the target stimulus located at retinotopic location A. Then, brief memory reactivations of only five trials each were conducted on separate days at location A. Generalization was later tested at retinotopic location B. A control group performed the task in retinotopic location A and then at location B without memory reactivations.

## Methods

### Participants

Fifty-eight healthy adults aged 18–40 years (9 males, average age 24.13 years SD = 3.71), participated in the study. All Participants provided written informed consent to participate, and the procedure was conducted in accordance with a protocol approved by Tel Aviv University’s Ethics Committee. All methods were performed in accordance with the relevant guidelines and regulations. The participants had normal or corrected-to-normal vision, were not video gamers^59^, did not participate in other visual experiments during the study, and reported at least 6 h of sleep the night before each experimental session (performed during daytime). Two participants were not included in the analysis due to an extreme encoding threshold (z-score > 2.5).

### Stimuli and task

**Fig. 1:**
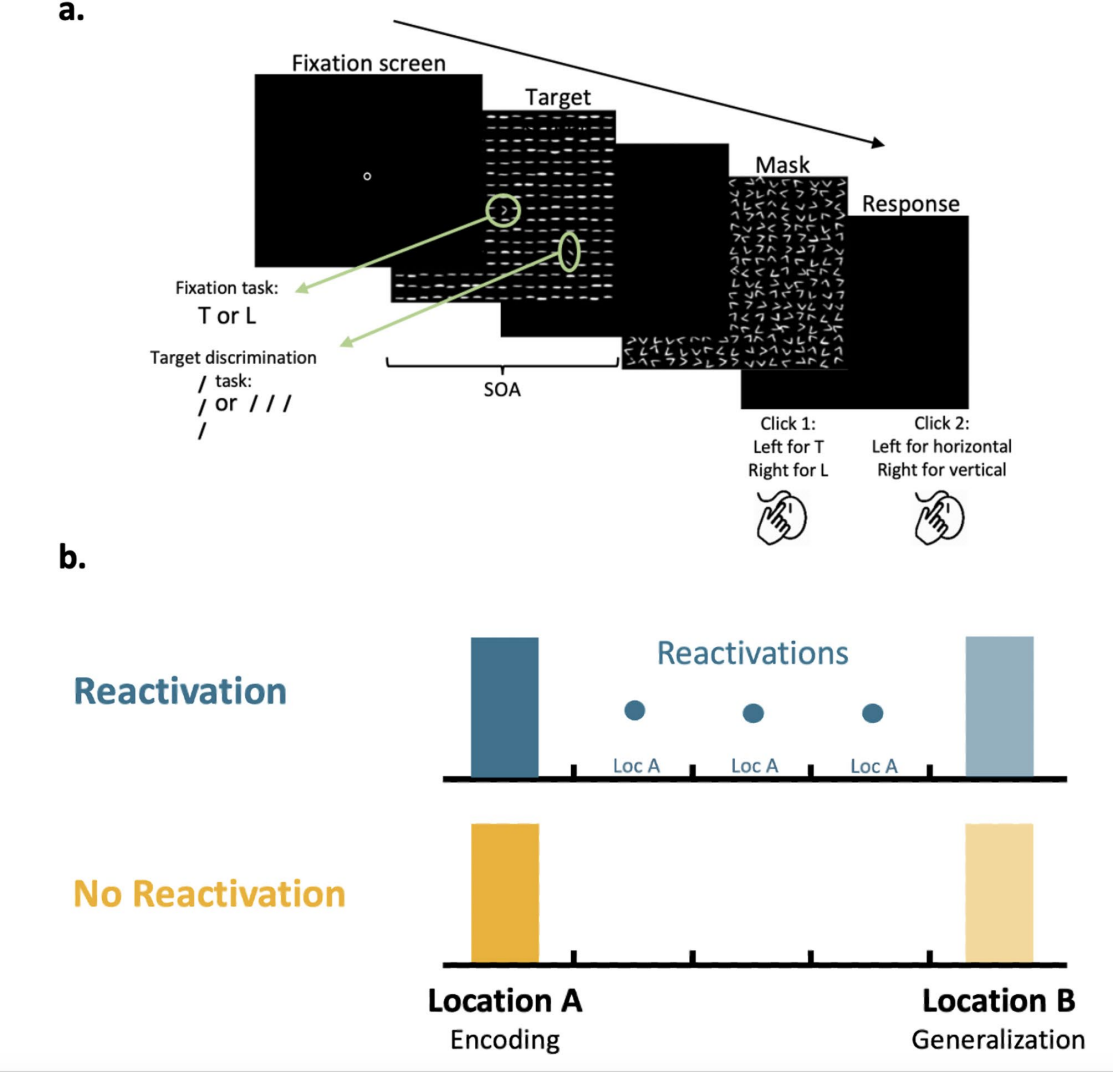
Texture discrimination task (TDT) and experimental procedure. (**a**) TDT example trial. Observers were required to discriminate between a horizontal or vertical orientation of a peripheral target consisting of three diagonal bars appearing for 10ms. Green circles and arrows are presented for illustration purpose and did not appear in the task. Fixation was enforced by a forced-choice letter discrimination task (rotated T or L) at the center of the display and was followed by auditory feedback for incorrect discrimination. The target-to-mask asynchrony (SOA, measured from the target’s onset to the mask’s onset) varied within the session to obtain a psychometric curve, from which the SOA discrimination threshold was derived. (**b**) Experimental procedure. An encoding session with the target stimulus appearing on location A (LR or LL) was performed by the Reactivation (blue) and No Reactivation (yellow) groups. The Reactivation group then performed three sessions in which the perceptual skill memory was briefly reactivated with only five reactivation trials on location A (represented by blue dots). Generalization was measured for the Reactivation and the No Reactivation groups nine days following the encoding session. Sessions were performed on separate days.

Participants performed a standard texture discrimination task, TDT^1^, with a 10ms target screen, followed by a 100ms mask (Fig. 1a). Observers had to discriminate whether a target array consisting of three diagonal bars (appearing 5.46° from the center of the visual field, either in the lower right (LR) or the lower left (LL) quadrant) was horizontal or vertical, and responded by pressing one of the two mouse buttons. Consistent with previous studies using the texture discrimination task, no feedback for correct or incorrect responses was provided^10–12,37,56–58,60^. The target stimulus was embedded in a background consisting of horizontal bars (19 × 19 bars, 0.57° × 0.04° spaced 0.86° apart, 0.04° jitter). Fixation was enforced by a forced-choice letter discrimination task, in which observers had to discriminate whether a rotated letter, presented in the center of the screen, was a T or an L, by pressing one of the two mouse buttons, with auditory feedback for incorrect discrimination. As maintaining fixation is fundamental for the peripheral target task, studies have designed and executed this paradigm so that participants are instructed to provide their responses to the fixation task first, highlighting the importance of fixation. Accordingly, participants provide their response for the fixation task (central T or L) first, and then respond to the target task (peripheral horizontal or vertical bars). Both responses are given while a black screen is presented. Importantly, as the task has been designed to enforce fixation at the center of the display^1^, auditory feedback for errors is provided only for the central fixation task and participants with low fixation task performance are excluded^61^. In the current study, the fixation performance was high, 94.9% ± 0.01 (Reactivation group) and 94.8% ± 0.01 (No Reactivation group) at the SOA closest above the threshold of the peripheral target at the encoding session, and also in the reactivation trials, 92.6% ± 0.06, pointing to compliance with the fixation instructions. In addition, fixation performance increased in the second generalization session (97.9% ± 0.01 for the Reactivation group and 96.5% ± 0.001 for the No Reactivation group), impying that participants in both groups maintained fixation on the central task.

Display size was 15.4° × 3 15.1°, viewed from 108 cm on a 20-in (50.8-cm) CRT HP p1230 monitor, refresh rate 100 Hz, mean texture luminance 84 cd/m^2^. The time interval between the target stimulus and the mask (stimulus-to-mask onset asynchrony, SOA, measured from the onset of the target to the onset of the mask) ranged from 40 ms to 340 ms (40, 60, 80, 100, 120, 140, 160, 180, 200, 220, 240, 260, 300, and 340 ms) and was pseudo-randomized across trials. Each block consisted of 2 trials per SOA (for a total of 252 trials over nine blocks). To familiarize the participants with the task at each retinotopic location, pre-training blocks consisting of 10 trials were conducted prior to the performance in the encoding and generalization sessions^49,55,61,62^. Of note, pre-training at location A was provided only before the encoding sessions, while pre-training at location B was provided only before the generalization sessions. Thus, participants had no prior exposure to location B before the day of the generalization measurement. These pre-training blocks were conducted initially with an SOA of 500 ms and then repeated with an SOA of 340 ms until subjects achieved 90% accuracy. A maximum of 10 blocks overall was provided, after which participants who did not reach this criterion in the encoding session did not continue to the experiment (mean number of pre-training blocks = 2.8 ± 1.2). Three participants did not reach this criterion. Pre-training at the encoding session was followed by an additional short familiarization block of 1 trial per SOA, without any minimum performance requirement. This block was designed to familiarize participants with the general temporal variance of the task, and therefore performed only before the initial encoding session. Then, participants continued to the main session (nine blocks as described above). To ensure reliable measurements in both locations, participants were required to reach above 80% correct responses on the three longest SOAs and above 0.8 finger errors (see below). All sessions were performed in a dark, quiet room.

## Experimental design

The memory of twenty-seven participants was encoded in a standard texture discrimination task (TDT) session with the target stimulus presented in retinotopic location A (either LR or LL; encoding session; Fig. 1b, Reactivation group), during which the discrimination threshold was measured. Participants then returned for three sessions on separate days, during which the encoded memory was reactivated with only five near-threshold memory reactivation trials^49,55^. Reactivation trials were set individually at the SOA given in the initial session that was closest above threshold. For example, for a participant with a 126 ms threshold, the reactivation SOA would be set to 140 ms. The average reactivation SOA was 131.8 ± 6.5 ms. (for threshold measurement see Data analysis section), consistent with previous studies^47,49,55^. Generalization was later tested, using the same SOAs, at retinotopic location B (LL or LR respectively; generalization session). All experimental sessions took place every other day (or with a 2-day interval on weekends).

Additional twenty-nine participants performed an encoding session at retinotopic location A (either LR or LL; Fig. 1b) and a generalization session on retinotopic location B (LL or LR, respectively) with no additional sessions between them (No Reactivation group). To maintain a similar generalization time interval between groups, the generalization session was performed ~ 9 days after the encoding session in both groups (mean intervals and SE of 9.0 ± 0.0 days for the Reactivation group and 9.1 ± 0.1 days for the No Reactivation group). Note that performance at location B was measured only at the test session, nine days following the encoding. This was consistently done for both groups. All sessions were performed during daytime.

### Data analysis

The individual visual thresholds were calculated for the encoding and the generalization sessions using the standard Weibull fit for the psychometric curve with slope β and an additional finger-error parameter 1-*p* yielding the function^18,63^:

$$\:Y\left(x\right)=p\left(1-\frac{1}{2}{\text{e}}^{-{\left(\frac{x}{T}\right)}^{{\upbeta\:}}}\right)+\frac{1-p}{2}$$

Where *x* is the SOA, *Y* is the success ratio (in the closed interval [0,1]) of target discrimination for a given SOA, and T is the threshold for the curve, defined as the SOA for which 81.6% of responses were correct when *p* = 1.

To first test for baseline differences in encoding thresholds, an independent sample t-test was conducted with location A threshold as the dependent variable and group as the independent variable.

To evaluate generalization, the difference between the discrimination threshold of the retinotopic locations (location A – location B; generalization score) was calculated for each participant. Then, one-way analysis of covariance (ANCOVA) with the experimental group as a fixed factor and threshold difference as the dependent variable was used to compare generalization between groups. The initial encoding threshold and the order of the retinotopic locations were included as covariates. To assess generalization amplitude, a post hoc paired sample t-test was conducted for each group.

To evaluate the correlation between performance in reactivation trials and the generalization score among participants showing generalization following memory reactivations, reactivation performance metrics were calculated as a percentage of correct responses out of the total 15 reactivation trials. Based on previous studies, we hypothesized a positive correlation between performance and reactivation trials^50,55^. To test this hypothesis, a one-tailed Pearson’s r coefficient was calculated.

To compare reactivation-induced generalization (improvement from location A to B) with same-location reactivation-induced learning gains measured in a previous study^55^ (improvement from location A to A), a one-way ANCOVA was performed with group as the fixed factor, gain (generalization or learning) as the dependent variable, and the initial threshold as a covariate.

## Results

We first verified that there were no baseline differences (*t*_*54*_ = 0.13, *P* = 0.90; *B*_*01*_ = 3.68 ± 0.009) between the Reactivation (mean threshold and SE = 122.99 ± 6.27 ms, *n* = 27) and No Reactivation (124.13 ± 6.47, *n* = 29) groups.

**Fig. 2:**
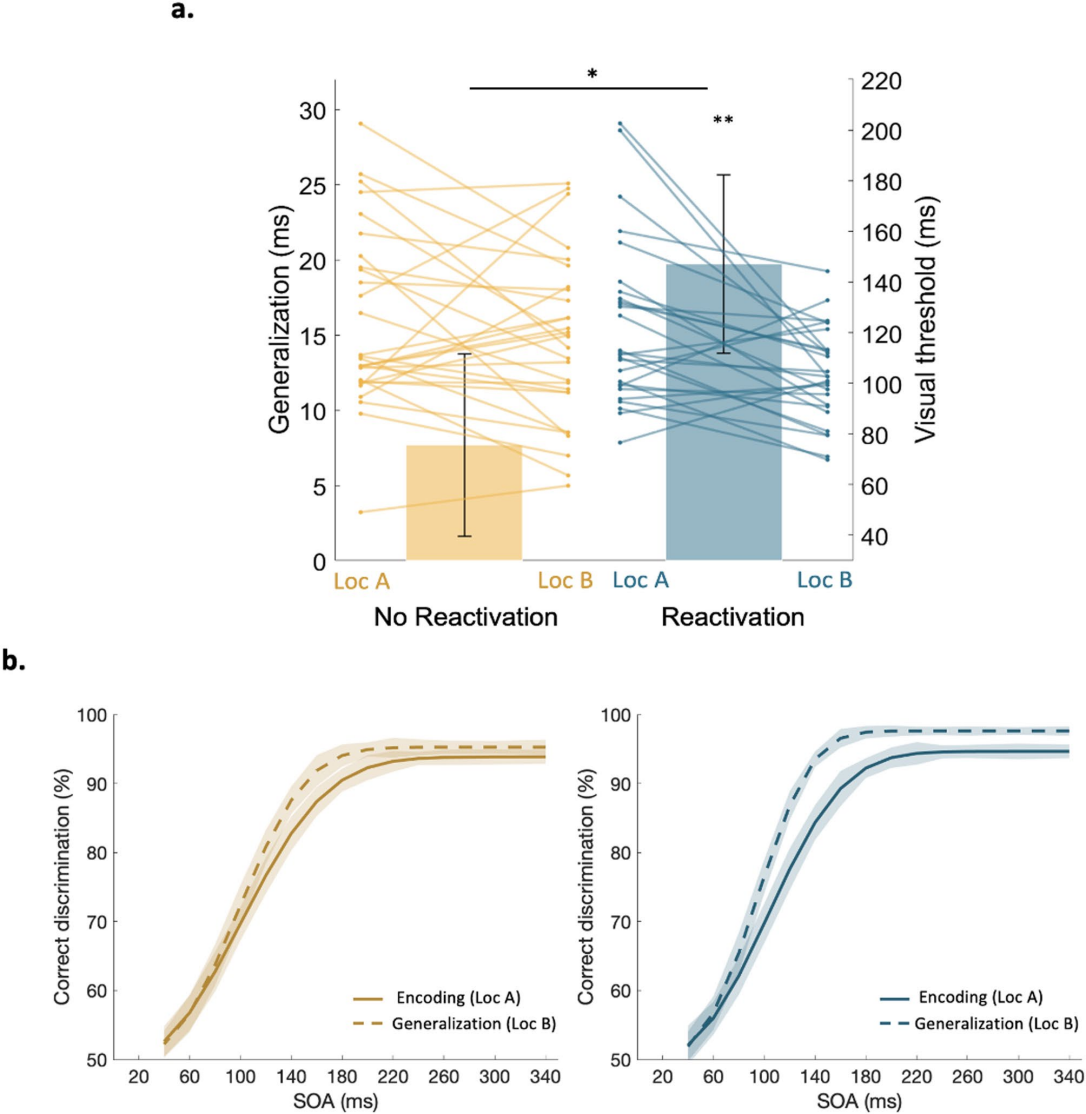
Generalization. (**a**) Mean generalization score (main bars, left Y axis) and individual visual thresholds of locations A and B (thin lines, right Y axis), indicating that generalization is enhanced with reactivation-induced learning (blue) compared to no memory reactivations (yellow). Error bars represent SE. * represent p value < 0.05, **p < 0.01. (**b**) Mean psychometric curves of the encoding (solid line) and the generalization (dashed line) sessions for the No Reactivation (yellow) and the Reactivation (blue) groups. A leftward shift indicates enhanced discrimination thresholds (see Methods, Data Analysis, and Supplementary Figure S1). Transparent envelopes represent SE.

A significant difference in generalization was observed between the Reactivation (mean generalization 19.72 ± 6.05 ms) and the No Reactivation (7.68 ± 5.92 ms) groups (*F*_*1,52*_ = 4.38, *P* = 0.04, Fig. 2a, b). Post-hoc analyses confirmed significant generalization in the Reactivation group (*t*_26_ = 3.33, *P* = 0.003) and no generalization in the No Reactivation group (*t*_28_ = 1.27, *P* = 0.21). These results suggest that memory reactivation enables generalization of offline learning mechanisms.

Interestingly, following memory reactivations, a linear correlation was not observed between performance in reactivation trials and generalization score (Pearson’s *r* = 0.12, *P* = 0.27). Thus, while in our previous studies it has been shown that reactivation performance correlated with learning^50,55^, it did not correlate with generalization, suggesting that reactivation-induced learning and generalization mechanisms are not identical.

**Fig. 3:**
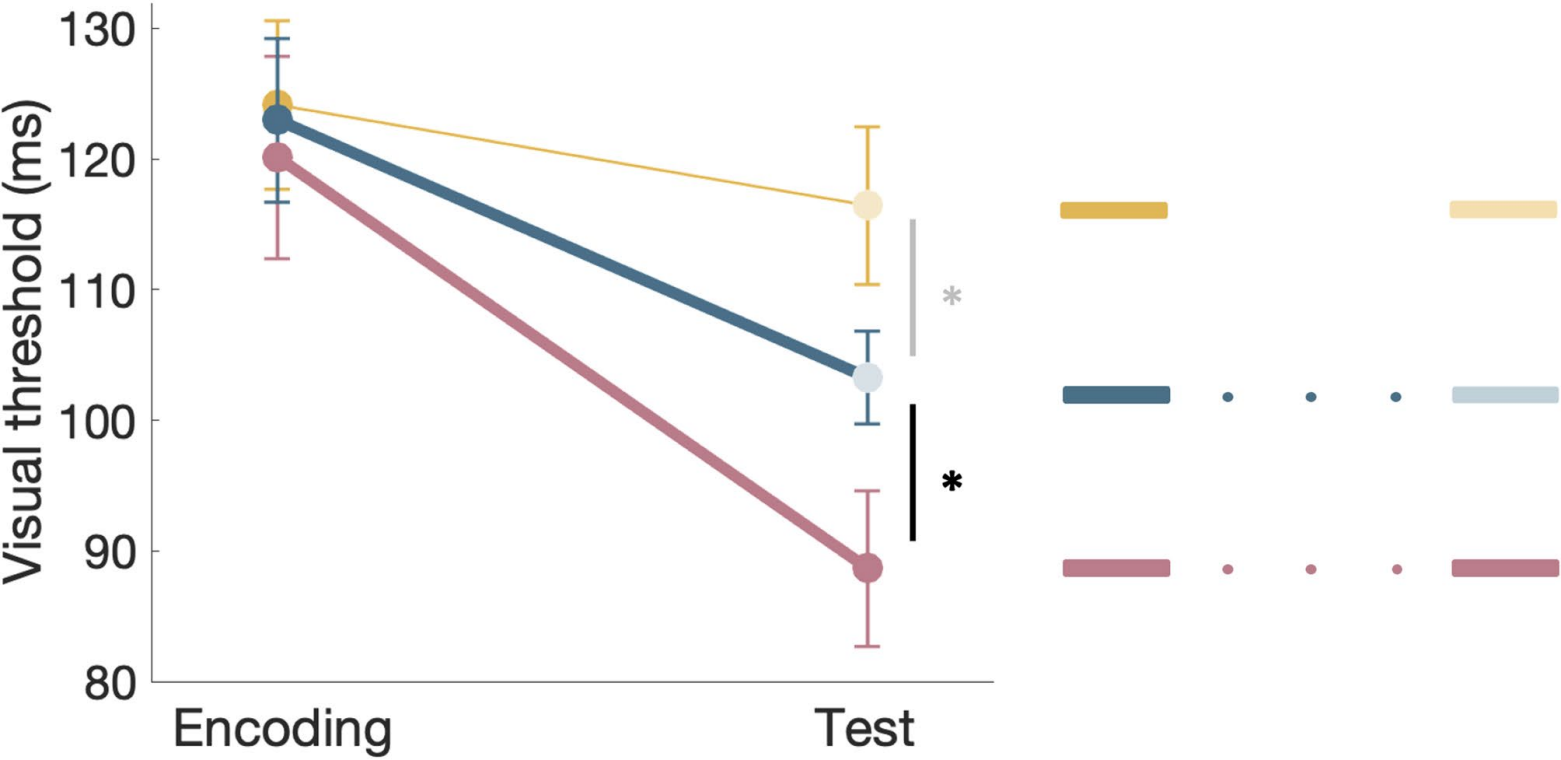
Reactivation-induced learning and generalization. Visual thresholds before (Encoding, target location A) and after learning (Test, target location A solid marker, target location B light marker). Pink - improvement in one retinotopic location following reactivation-induced learning, taken from our previous study using the same task^55^; Blue - improvement following reactivation-induced generalization; Also presented in thin yellow – generalization without reactivation. Experimental protocols are outlined on the right, with solid rectangles representing target location A and light rectangles representing target location B. * *p* < 0.05. Error bars represent SE.

Comparing reactivation-induced generalization gains (19.72 ± 6.05 ms, improvement from location A to B) with same-location gains in reactivation-induced learning (31.46 ± 5.59 ms, improvement from location A to A, measured in our previous study using the same task^55^) reveals a significant difference (F_1,44_ = 5.80, *P* = 0.020), suggesting that only part of the learning is generalized to the untrained location (Fig. 3).

## Discussion

The current study was designed to examine whether perceptual learning induced by memory reactivations, improves generalization of offline learning mechanisms. The results showed that generalization was remarkably enhanced following memory reactivations. In contrast, minimal generalization was evident without reactivations. The efficiency of learning can be expressed in various aspects. Generalization of learning is one such aspect, extending the learning scope beyond the specific training conditions. Therefore, it is often a highly desirable outcome of the learning process.

Recent evidence suggests that perceptual learning, typically driven by practice-dependent plasticity and demanding repetitive exposure to a stimulus, can also be induced through a distinct pathway with brief stimulus exposures on separate days^49,55,64^, which function as memory reactivations of a previously encoded skill. Notably, reactivation per se does not act as additional training, for example, performing all reactivation trials on the same day does not result in reduced thresholds^49^. Therefore, the brief memory reactivations may generate subsequent offline cycles of memory modulation through reconsolidation^44,65,66^. Such reactivations mediate perceptual gains by recruiting enhanced engagement of higher-order attention and control brain regions^55^.

Efficient generalization following memory reactivations may arise from this significant engagement of higher-order regions^55^. Perceptual learning is often highly specific to the trained stimulus features^1,7,12–16,67^. Its neural mechanisms are predominantly attributed to changes in early visual areas, where neural coding shows specificity to simple visual features, such as orientation and location^11,12,23,24,36,47,68,69^. However, engagement of higher-order brain regions has been documented in perceptual learning^12,34–42^, suggested to mediate global aspects of the task and readouts from low-level regions, and indicating more complex learning mechanisms. The ability to generalize perceptual learning has been demonstrated under certain conditions^26–33,70^, including attentional manipulation^33^, double training^30^, removal of sensory adaptation^32^, and the association with higher-order brain regions that are not specific to simple visual features^71–73^. Moreover, studies have shown that generalization of learning is associated with parietal cortex activity^57,74,75^. Therefore, recent evidence indicating that reactivation-induced perceptual learning is mediated by enhanced engagement of higher-level brain resources, including the parietal cortex^55^, may also explain generalization effects, as shown here.

Although remarkably significant, reactivation-induced generalization gains were lower in magnitude compared to same-location gains in reactivation-induced learning measured in our previous study^55^, and while reactivation performance correlated with learning^50,55^, it did not correlate with generalization. Of note, such comparisons should be interpreted cautiously since they are performed across studies. We did not incorporate both location conditions retested within the same design in light of previous findings^61^, suggesting that repeated measurements of location A performance may in itself influence generalization. Nevertheless, these findings may suggest that the reactivation-induced learning and generalization mechanisms are not identical, and that while learning is partially generalized, some of its aspects may remain specific to the trained features. Previous studies that documented learning transfer, trained participants with one feature (e.g., contrast) at one location, and a different feature (e.g., orientation) at a second location, showing generalization of the original feature to the second location^30,31,70^. The authors suggested that such generalization may result from central learning (in higher-level brain regions) transferred to the second location after its spatial attention (which is not feature-specific) improved^30^. Other studies have also suggested that learning and generalization may be facilitated by the existence of higher-level components and their interactions with early regions^23,71,76,77^. Another study found that when a task was performed in location B immediately after location A, at the same session, it led to enhanced generalization and integrated learning across conditions, possibly due to the formation of a unified network following simultaneous consolidation of the two locations^61^. These approaches empirically pre-define and act upon the new retinotopic locations where learning is expected to be observed, potentially restricting generalization to these regions. However, memory reactivation may enable generalization not only to a single pre-defined additional location, but rather to any new location, pointing to a global generalization mechanism.

Beyond the association between early visual areas and specificity in perceptual learning, research has shown that specificity is increased with prolonged training^56,78–80^ which may lead to overfitting^81^. Specifically, Jeter et al.^80^ showed that the more training sessions one undergoes, the more specific the learning becomes, reducing its generalization. This interpretation may also be consistent with the results showing generalization in reactivation-induced learning with minimal stimulus exposure.

A limitation of this study is that it did not examine generalization gains following repetition practice. Previous research has shown that reactivation-induced learning gains were comparable to repetition practice^49^ and a replication of these results has been further documented^55^. However, while repetition practice gains have been consistently shown to be highly specific, with minimal generalization^1,7,12–16,67^, the current study employed a direct within-study comparison to a no-reactivation rather than to a repeated-practice condition.

In addition, an alternative hypothesis could suggest that participants have learned a more effective fixation point between the central fixation task and the peripheral task. It is important to note that the encoding trials in both groups, as well as the reactivation trials, were all performed in location A. However, only the Reactivation group showed enhancement of location B performance. Therefore, such a fixation point (between the central and the peripheral task) for performing the peripheral task at location A during encoding and reactivation trials, is not predicted to be efficient for performing the peripheral task at the new location B. In addition, this alternative hypothesis would predict that learning in the texture discrimination task should generalize to new locations, however, this is often not the case and learning in the texture discrimination task is commonly highly specific^1,7,12–16^. Nevertheless, it may be useful to leverage eye-tracking paradigms in future studies in the field. This may also potentially reduce the impact of dual-tasking on working memory and cognitive load.

The results of the current study indicate that brief memory reactivations enable generalization of offline visual perceptual learning mechanisms, further enhancing learning efficiency. Such benefits of learning and the understanding of their underlying mechanisms may have important implications for optimizing learning and skill acquisition across domains, and in rehabilitation requiring efficient re-learning and generalization.

## Electronic supplementary material

Below is the link to the electronic supplementary material.

Supplementary Material 1

## Data Availability

The datasets used and analyzed during the current study are available from the corresponding author upon reasonable request.
