# Supplementary material for: Brief memory reactivations enable generalization of offline visual perceptual learning mechanisms
Source: Sci Rep. 2025 Jul 1;15:22137. doi: 10.1038/s41598-025-06564-y (PMC12219644; doi:10.1038/s41598-025-06564-y)
Supplement: Supplementary file 1 — Supplementary Material 1 [file 41598_2025_6564_MOESM1_ESM.docx]

**Supplementary**

**Brief memory reactivations enable generalization of offline visual perceptual learning mechanisms**

Taly Kondat^1,2^, Yuka Sasaki^3^, Takeo Watanabe^3^, Nitzan Censor^1,2^

^1^Sagol School of Neuroscience, Tel Aviv University, Tel Aviv 69978, Israel

^2^School of Psychological Sciences, Tel Aviv University, Tel Aviv 69978, Israel

^3^Department of Cognitive and Psychological Sciences, Brown University, Providence, USA

**
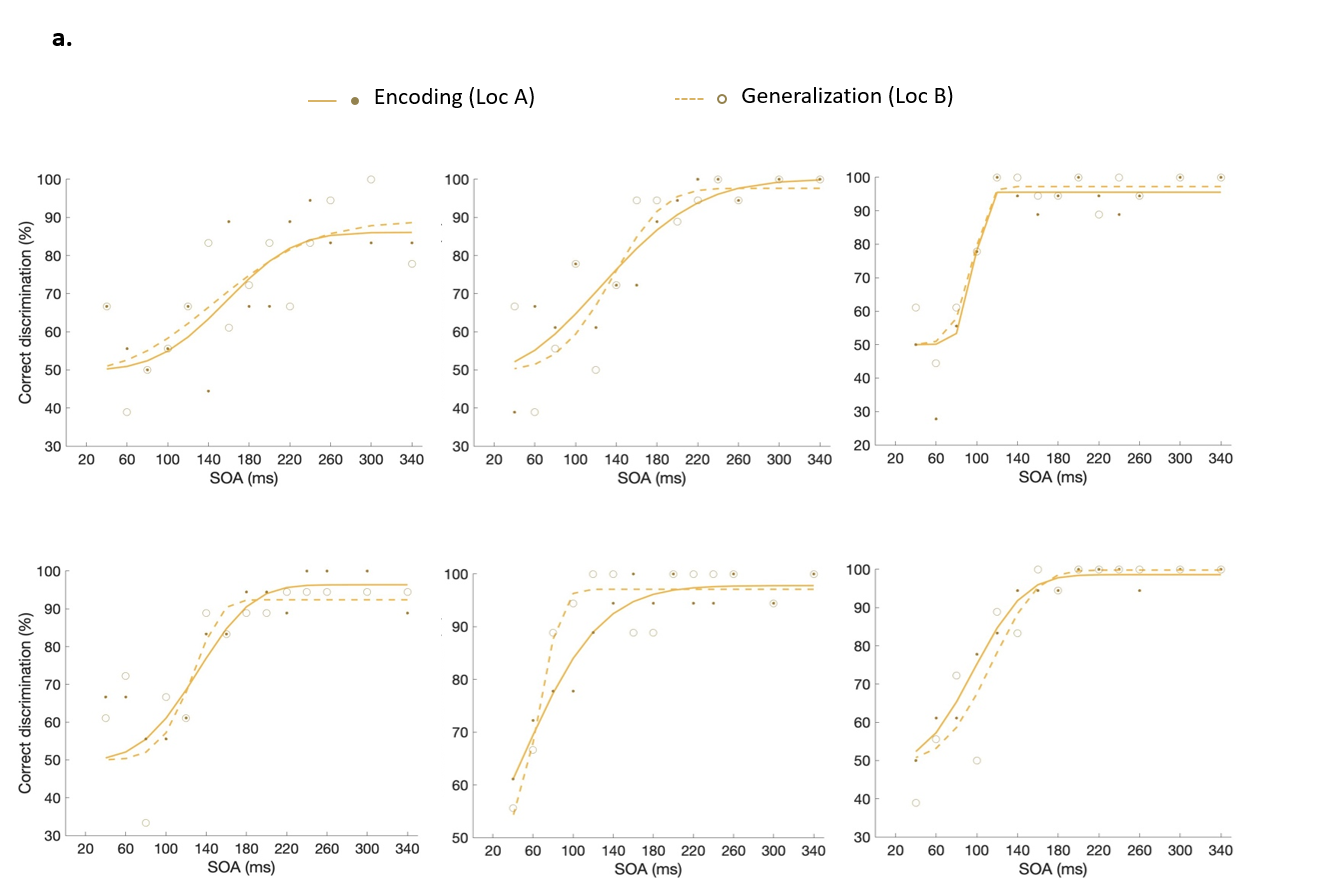
**


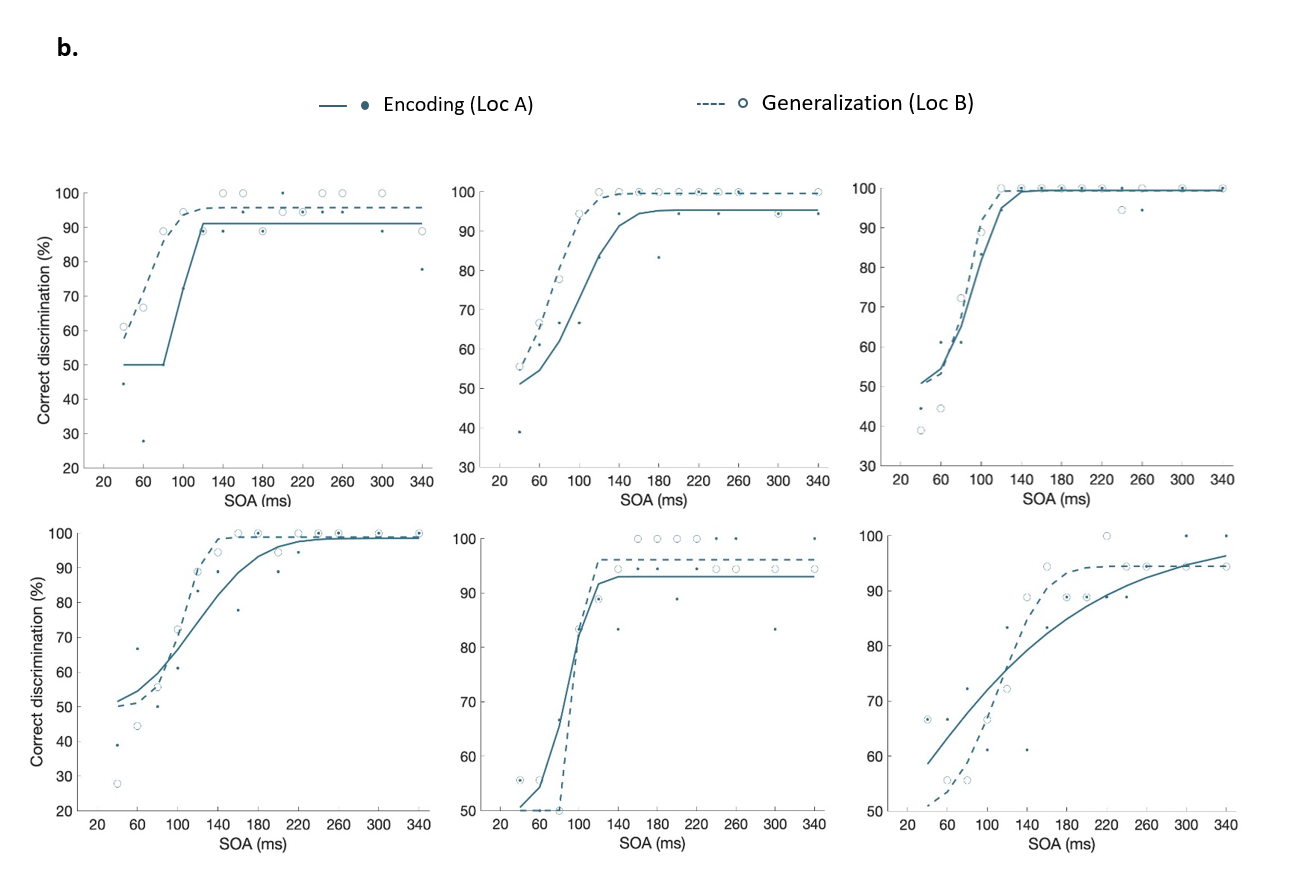


**Figure S1. Examples of individual participants psychometric curves. (a) No reactivation group participants and (b) Reactivation group participants. Solid dots and open circles present the data points of the encoding and generalization sessions, respectively. Solid and dashed lines present the fitted psychometric curve. A leftward shift indicates enhanced discrimination thresholds (see Methods, Data Analysis, and main Figure 2b).**
